# High-degree centrum semiovale-perivascular spaces are associated with development of subdural fluid in mild traumatic brain injury

**DOI:** 10.1371/journal.pone.0221788

**Published:** 2019-09-04

**Authors:** Hae-Won Koo, Minkyung Oh, Hyung Koo Kang, Yung Ki Park, Byung-Jou Lee, Seong Rok Han, Sang Won Yoon, Chan Young Choi, Moon-Jun Sohn, Chae Heuck Lee

**Affiliations:** 1 Department of Neurosurgery, Ilsan Paik Hospital, College of Medicine, Inje University, Neuroscience, Radiosurgery and Adaptive Hybrid Neurosurgery Research Center, Goyang, Korea; 2 Department of Pharmacology, College of Medicine and Clinical Trial Center, Busan Paik Hospital, Inje University, Busan, Korea; 3 Division of Pulmonary and Critical Care Medicine, Department of Internal Medicine, Ilsan Paik Hospital, College of Medicine, Inje University, Goyang, Korea; University Hospital Zurich, SWITZERLAND

## Abstract

**Background:**

Severe centrum semiovale perivascular spaces (CSO-PVSs) are associated with the onset of brain atrophy and dementia. This study explored the relationship between severity of CSO-PVS and development of subdural fluid (SDF) in patients with mild traumatic brain injury (TBI), with the aim of investigating independent radiological risk factors for development of SDF.

**Methods:**

The study cohort comprised 222 patients with a mean age of 51 years (64.0% men) who presented with mild TBI from January 2013 to November 2016. In this study, mild TBI was defined as a Glasgow Coma Scale (GCS) of ≥ 13, Post-Traumatic Amnesia (PTA) of <1 day, and Loss of Consciousness (LOC) of <30 minutes. The severity of CSO-PVS was categorized as low or high-degree.

**Results:**

Among the 222 enrolled patients, 38 (17.1%) and 90 (40.5%) had high-degree PVS in the basal ganglia (BG) and centrum semiovale, respectively. Compared with patients who did not develop SDF, the mean age of patients who developed SDF was significantly higher (47.41 years versus 60.33 years, P < 0.0001). The incidence of de novo SDF was significantly higher in men than in women (77.8% versus 59.5%, P = 0.0151). Patients who showed SDF on brain computed tomography at admission more frequently developed de novo SDF (68.5% versus 38.1%, P < 0.0001). In multivariate logistic regression analysis of risk factors, high-degree CSO-PVS, male sex, initial SDF on admission, and old age were independently associated with development of de novo SDF after mild TBI. In Cox proportional hazards models of risk factors for SDF-development free survival rate, high-degree CSO-PVS, old age, and initial subdural hemorrhage showed statistically significant differences.

**Conclusions:**

Our study might help neurosurgeons determine the frequency of brain CT or the duration of follow-up for patients who present with mild TBI with high-degree CSO-PVS.

## Introduction

Traumatic brain injury(TBI) can be classified as mild, moderate, or severe, and approximately 75% of TBI patients present with mild TBI[1]. The most common classification system for the severity of TBI is the Glasgow Coma Scale (GCS) score[2]. Generally, TBI with a GCS of ≥13 is regarded as mild, whereas ≤12 is moderate to severe. To overcome limitations in predicting the outcomes and prognosis based on GCS score, however, current approaches involve the use of three criteria: duration of post-traumatic amnesia (PTA), loss of consciousness (LOC), and GCS score[3, 4].

Post-traumatic subdural fluid (SDF) collection comprises accumulation of cerebrospinal fluid (CSF) in the subdural space, occurring in 4–6.6% of head-injured patients; it is considerably more common in older patients[5, 6]. Unlike patients with moderate to severe TBI, most patients with mild TBI rarely present with SDF or subdural hemorrhage (SDH) on admission. However, during follow-up management for patients with mild TBI, many neurosurgeons have encountered radiologic findings that include increased SDF or the transition to chronic subdural hematoma (CSDH). As a greater proportion of society enters older age, the frequency of CSDH is likely to increase further.

Several studies have reported development of CSDH following traumatic SDF[7–10]. Although patients with mild TBI occasionally show increased SDF or transition to CSDH, the specific pathophysiology is not well known. There have been many studies regarding risk factors for subsequent development or recurrence of CSDH[5, 7–12]. Among these risk factors, old age-related brain atrophy has been identified as an important factor for development of CSDH.

Enlarged perivascular spaces (PVSs), or Virchow Robin spaces, are common radiologic findings with identical signal intensities to CSF in T2-weighted magnetic resonance (MRI) sequences, especially in elderly patients. The sizes and numbers of normally microscopic PVSs increase with advancing age; thus, PVSs appear on T2-weighted MRI as round or tubular hyperintensities in basal ganglia (BG) and centrum semiovale[13, 14]. Enlarged PVSs are associated with a variety of neuropsychiatric disorders, including small vessel disease[14, 15], intracranial atherosclerosis[16, 17], multiple sclerosis[18], dementia[14, 19, 20], depression[21], systemic lupus erythematosus[22] and various others[23–25].

The aim of the present study was to investigate independent radiological risk factors for the development of SDF. To the best of our knowledge, there has been no MRI analysis of PVS severity-related factors for development of CSDH. Therefore, we closely evaluated the relationship between severe enlarged PVSs and development of SDF.

## Materials and methods

### Study population and data collection

This was a retrospective single-center study of patients with mild TBI. Electronic database searches were used to identify consecutive TBI patients. In total, 1324 consecutive patients with TBI presented to our hospital from January 2013 to November 2016. Among them, patients were enrolled in the present study if they (1) had a brain MRI, including T2-weighted imaging; (2) did not have a history of previous trauma or diseases such as stroke, tumors, or degenerative disease (e.g., dementia or Parkinson’s disease). Among those patients that met these criteria, mild TBI was defined as follows: (1) LOC of approximately <30 minutes or (2) an initial GCS score of 13–15, and (3) PTA < 24 hours. Among 1324 patients with TBI, 892 (67.4%) presented with mild TBI and 432 (33.6%) presented with moderate to severe TBI. Among the 892 patients with mild TBI, 225 underwent brain MRI, including T2-weighted imaging. However, three patients could not be evaluated regarding PVS severity due to poor image quality.

Several basic demographic features and risk factors were assessed in mild TBI patients. These included age at admission, sex, hypertension, diabetes mellitus, hyperlipidemia, liver cirrhosis, history of anti-thrombotic agent use, operation for drainage of SDF, initial SDF of SDH on computed tomography (CT) at admission, white matter hyperintensity graded by the Fazekas scale (0–3), and high-degree of centrum semiovale perivascular spaces (CSO-PVSs) and basal ganglia perivascular spaces (BG-PVSs). Hypertension was defined on the basis of prior diagnosis and the intake of antihypertensive drugs

The Institutional Review Board of Ilsan Paik Hospital approved this study, including the review and publishing of information obtained from patient records (IRB no.: 2017-04-029). The requirement for informed consent was waived for the use of patient medical data, as all patient information was anonymized and de-identified prior to the analysis.

### CT and brain MRI acquisition and analysis

Most patients underwent follow-up brain CT every week for approximately 3 weeks after trauma, or at the onset of symptom or sign exacerbation. If SDF, SDH, or other type of traumatic intracranial hemorrhage was detected on initial brain CT, we closely monitored the patients. Brain CT was performed once per week routinely until development of SDF had stabilized. SDF diagnosis was based on published brain CT scan criteria of hypodense SDF collection after TBI, with a minimum distance between the skull and brain of at least 3 mm[26, 27].

Brain MRI was performed after SDF was stabilized on follow-up brain CT. All subjects had an axial T2-weighted fast spin-echo sequence (TR/TE, 4850/98 ms; flip angle, 90°; FOV, 220 mm; slice thickness, 5 mm; and slice gap, 6 mm) and a T2*-weighted gradient echo sequence (TR/TE, 800/26 ms; flip angle, 20°; FOV, 230 mm; slice thickness, 5 mm; and slice gap, 6 mm) based on data obtained using a 1.5-Tesla MRI scanner.

PVS severity was assessed and rated on axial T2-weighted MRI in accordance with recent published method[28, 29]. Enlarged PVSs were defined as small (<3 mm), sharply delineated structures of CSF signal intensity that followed the orientation of perforating arteries in T2-weighted MRI. After reviewing all relevant slices for the assessed anatomical area, the unilateral side of the slice with the highest number of PVSs was recorded. To confirm our hypothesis, PVS severity in the BG and centrum semiovale was categorized into low-degree (n ≤ 20) and high-degree (n > 20) groups. PVS severity was interpreted by two independent board-certified neurosurgeons. In cases of disagreement, we sought consensus between the two observers and a third interpreter[29].

### Statistical analysis

Patients were dichotomized into two groups based on de novo SDF collection. We studied associations between development of SDF or SDH and independent variables, including demographic features and radiological findings. For patients with de novo SDF, subgroup analysis was performed for risk factors of operation.

The chi-squared test or Fisher’s exact test were used to compare categorical variables, whereas the *t*-test or Mann-Whitney U test were used to compare continuous variables. Multivariable logistic regression with stepwise selection was used to identify independent clinical and radiologic risk factors associated with the development of de novo SDF and operations in patients with mild TBI. Cohen’s kappa statistic was calculated to test interrater reliability for evaluation of CSO-PVS and BG-PVS severity on T2-weight MRI. We evaluated risk factors that affected the duration for development of de novo SDH using Kaplan-Meier method. In patients who did not show development of SDF, the duration of follow-up was limited to 90 days. A P-value <0.05 was considered to be statistically significant. SAS 9.4 (SAS Institute, Cary, NC, USA) was used for statistical analysis.

## Results

A total of 222 consecutive patients with mild TBI satisfied the inclusion criteria for this study. These patients had a mean age of 51 years and were 64.0% men. All patients presented with several radiological findings: 66 showed minimal SDH, 56 had multiple focal brain contusions, 35 had single minimal cerebral contusions, 12 had traumatic subarachnoid hemorrhage, 11 had minimal epidural hematoma, and 42 had simple concussion. Mild TBI causes were as follows: slip in 66 (29.7%) cases, in-car accidents in 41 (18.5%) cases, out-of-car accidents in 22 (9.9%) cases, bicycle accidents in 34 (15.3%) cases, falling in 21 (9.5%) cases, assault in 16 (7.2%) cases, and by being struck by or against an object in 22 (9.9%) cases. The mean GCS score at admission was 14.83 ± 0.54 (range, 13–15); 202 of 222 patients (90.9%) had a score of 15. Thirty-eight (17.1%) and 90 (40.5%) patients had high-degree BG-PVS and CSO-PVS, respectively. The interrater kappa values were 0.74 and 0.78 for EPVS in the BG and the centrum semiovale, respectively.

Table 1 shows the demographic features of risk factors based on the development of de novo SDF among the patients in this study; 54 (24.3%) patients showed development of de novo SDF and 168 (75.7%) did not. Overall, the mean follow-up period was 71.86 ± 33.08 days (range, 7–90 days). The mean period for development of de novo SDF from trauma was 14.28 ± 17.18 days (range, 1–90 days). Thirty-nine cases (72%) of de novo SDF were diagnosed within fewer than 14 days.

**Table 1:** Analysis of risk factors for the development of de novo subdural fluid collection in mild traumatic brain injury patients.

Characteristics	Development (n = 54)	Non-development (n = 168)	*P-value*
Age, y, mean (±SD)	60.33 ± 16.67	47.41 ± 18.49	*<0*.*0001*
Sex, male, n (%)	42 (77.8)	100 (59.5)	*0*.*0151*
High-degree CSO-PVS, n (%)	40 (74.1)	50 (29.8)	*<0*.*0001*
High-degree BG-PVS, n (%)	16 (29.6)	22 (13.1)	*0*.*005*
Initial subdural fluid	37 (68.5)	64 (38.1)	*<0*.*0001*
Glasgow Coma Scale, n (%)			*0*.*505*
13	6 (11.1)	11 (6.5)	
14	1 (1.9)	2 (1.2)	
15	47 (87.0)	155 (92.3)	
Surgery	12 (22.2)	0 (0)	*<0*.*0001*
Hypertension, n (%)	18 (33.3)	38 (22.6)	*0*.*1203*
Diabetes, n (%)	16 (29.6)	19 (11.3)	*0*.*0014*
Liver cirrhosis, n (%)	3 (5.6)	4 (2.4)	*0*.*2455*
Anti-thrombotic agent, n (%)	8 (14.8)	18 (10.7)	*0*.*4236*
WMH, Fazekas grade			*0*.*0203*
0	2 (3.7)	20 (11.9)	
1	39 (72.2)	123 (73.2)	
2	12 (22.2)	15 (8.9)	
3	1 (1.9)	9 (5.4)	

SD, standard deviation; CSO-PVS, centrum semiovale perivascular space; BG-PVS, basal ganglia perivascular space; WMH, white matter hyperintensity

The mean age of patients who developed SDF was significantly higher than that of patients who did not (60.33 years versus 47.41 years, P < 0.0001). The incidence of de novo SDF after mild TBI was significantly higher in males (77.8% versus 59.5%, P = 0.0151). High-degree CSO-PVS and BG-PVS were more frequent among patients who developed SDF than among those who did not (74.1% versus 29.8% for CSO-PVS, P < 0.0001; 29.6% versus 13.1% for BG-PVS, P = 0.005). Patients who showed SDF on brain CT at admission more frequently developed de novo SDF (68.5% versus 38.1%; P < 0.0001). Among vascular risk factors, diabetes mellitus was more frequent among patients who developed SDF (29.6% versus 11.3%, P = 0.0014). The development group showed more frequent moderate to severe white matter hyperintensity (Fazekas 2 or 3). There were no statistical differences in GCS score, anti-thrombotic agent use, hypertension history, or liver cirrhosis between the two groups.

In multivariate logistic regression analysis of risk factors, high-degree CSO-PVS, male sex, initial SDF on admission, and old age were independently associated with development of de novo SDF after mild TBI [Table 2]. Table 3 shows the characteristics of risk factors with regard to surgery among the patients in this study. Twelve (5.4%) patients who underwent surgical treatment by burr hole drainage were men. The mean age of patients who underwent surgery was significantly higher than that of patients who did not (66.17 years versus 49.66 years, P = 0.0034). High-degree CSO-PVS and BG-PVS were both more common in the surgery group than in the non-surgery group (75.0% versus 39.0% for CSO-PVS, P = 0.0138; 41.7% versus 15.7% for BG-PVS, P = 0.0203). More patients in the surgery group demonstrated SDF on brain CT at admission (75.0% versus 43.8%, P = 0.0348).

**Table 2:** Multivariate analysis of risk factors for development of de novo subdural fluid collection.

Variables	OR	95% CI	p-value
High-degree of CSO-PVS	5.242	2.52–10.91	<0.0001
Male sex	2.33	1.05–5.15	0.0377
Initial subdural fluid	2.32	1.1–4.89	0.0272
Age	1.031	1.01–1.05	0.006

CSO-PVS, centrum semiovale perivascular space; OR, odds ratio; CI, confidence interval

**Table 3:** Characteristics of mild traumatic brain injury patients according to surgical treatment.

Operation (Yes or No)	Yes (n = 12)	No (n = 210)	*P*-value
Age, y, mean (± SD)	66.17 ± 11.87	49.66 ± 18.77	0.0034
Sex, male, n (%)	12 (100)	130 (61.9)	0.0075
High-degree CSO-PVS, n (%)	9 (75.0)	82 (39.0)	0.0138
High-degree BG-PVS, n (%)	5 (41.7)	33 (15.7)	0.0203
Initial subdural fluid	9 (75.0)	92 (43.8)	0.0348
Glasgow Coma Scale, n (%)			0.5337
13	0 (0)	17 (8.1)	
14	0 (0)	3 (1.4)	
15	12 (100)	190 (90.5)	
Hypertension, n (%)	3 (25.0)	53 (25.2)	0.9778
Diabetes, n (%)	3 (25.0)	32 (15.2)	0.3713
Liver cirrhosis, n (%)	1 (8.3)	6 (2.9)	0.2911
Anti-thrombotic agent, n (%)	2 (16.6)	24 (11.4)	0.5878
WMH, Fazekas grade			0.4972
0	1(8.3)	21(10.0)	
1	8 (66.7)	154 (73.3)	
2	3 (25.0)	24 (11.4)	
3	0 (0)	10 (4.8)	

SD, standard deviation; CSO-PVS, centrum semiovale perivascular space; BG-PVS, basal ganglia perivascular space; WMH, white matter hyperintensity

In multivariate logistic regression analysis of risk factors, male sex and old age were independently associated with surgery for de novo SDF after mild TBI [Table 4].

**Table 4:** Multivariate analysis of risk factors for operation after mild traumatic brain injury.

Variables	OR	95% CI	p-value
Male sex	8.045	1.01–64.57	0.0497
Age	1.067	1.02–1.11	0.0021

OR, odds ratio; CI, confidence interval

**Fig 1:**
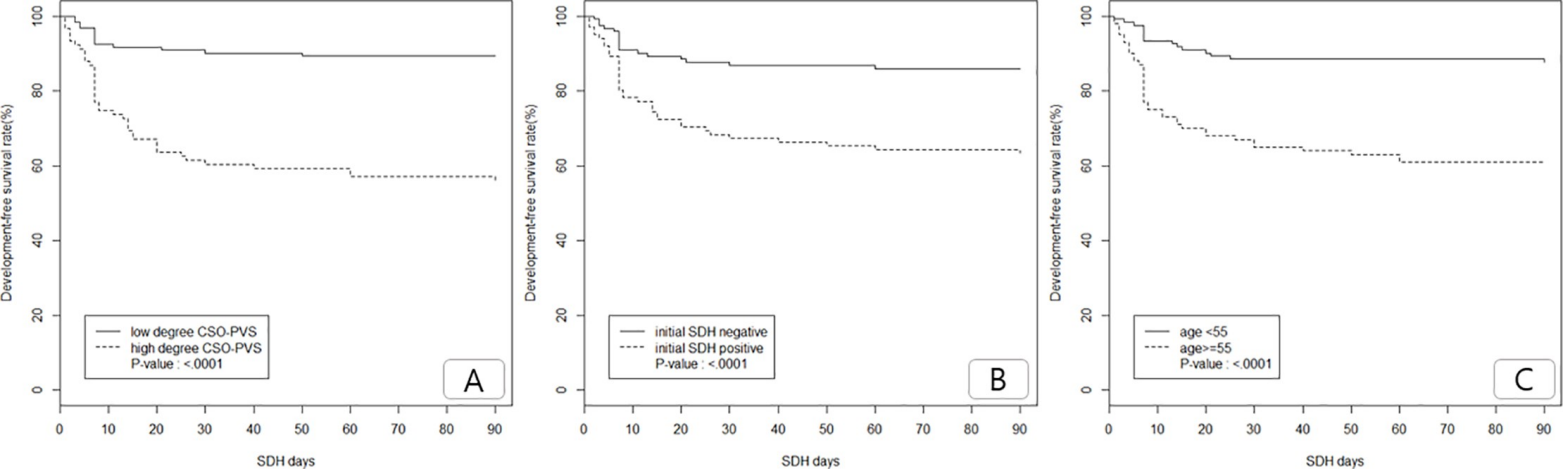
Subdural fluid-development free survival rate according to the severity of centrum semiovale perivascular space (CSO-PVS) (A), initial subdural hemorrhage (SDH) presence (B) and old age (C).

In analysis using Kaplan-Meier method for risk factors of SDF-development free survival rate, high-degree CSO-PVS, old age, and initial SDH showed statistically significant differences (log-rank test: P < 0.0001 for all) [Fig 1].

## Discussion

Our study focused mainly on the role of PVS, a known radiological marker of brain degeneration, in patients who developed SDF or CSDH after mild TBI. There were two main findings in our study. First, high-degree CSO-PVS, male sex, initial SDF on admission, and old age (≥55 years) were independently associated with development of de novo SDF, whereas BG-PVS was not. Second, high-degree CSO-PVS, old age (≥55 years), and initial SDH significantly affected the time for development of de novo SDH.

Our study was undertaken with the hypothesis that the development of de novo SDF after mild TBI may be more common in patients with brain atrophy on MRI; however, there remains considerable controversy regarding the relationship between brain atrophy and PVS severity[15]. A few studies have shown that the severity of PVS was not associated with brain atrophy [30, 31]. However, in a recent study of stroke patients, brain atrophy was associated with the severity of BG-PVS [15, 32]. Additionally, enlarged CSO-PVS was associated with cognitive function in healthy elderly patients [19].

PVSs are defined as fluid-filled spaces that follow the typical course of a vessel as it travels through gray or white matter[33]. PVSs also act as pre-lymphatic drainage system for the removal of substances. As arteries stiffen with age, the amplitude of pulsation is reduced, and insoluble amyloid-β is deposited in the interstitial fluid drainage pathway; this impedes drainage of soluble amyloid-β further. Subsequently, these pathophysiologic courses can lead to CSO-PVS dilation [29].

The Kashima Scan Study suggested that PVS distribution may be useful for early detection and classification of small vessel diseases or hypertensive diseases in neurologically healthy populations[34]. The presence of BG-PVS may be a marker for pure hypertensive arteriopathy, whereas the presence of CSO-PVS might reflect both hypertensive arteriopathy and cerebral amyloid angiopathy (CAA)[34]. Notably, the severity of CSO-PVS is associated with CAA, which is related to atrophy or lesions in cortical or subcortical brain lobes [29]. Our study showed that a high-degree of CSO-PVS was more prevalent in patients who developed SDF or CSDH after mild TBI, suggesting that atrophy of cortical or subcortical areas of the brain may be closely related to the development of SDF.

Several clinical risk factors have been suggested to contribute to the development or recurrence of CSDH: old age, male sex, bilateral SDF, high and mixed density on brain CT, medical diseases with bleeding tendency, and brain atrophy[7, 11, 12].

Several studies have shown male preponderance for the development of SDF and CSDH[7, 35–38]. This tendency was also detected in our study, but remains controversial. An existing rationale for male dominance is men generally have greater exposure to moderate to severe traumatic injuries[7]. However, we included only patients who met mild TBI criteria in our study. Additionally, male sex was analyzed as an important risk factor for requiring surgical treatment after mild TBI. The severity of TBI may also be an important factor for the development of SDF; however, the causative mechanism or event that led to TBI could also constitute an important factor for male dominance. An analysis of the development of SDF or CSDH on the basis of the causative mechanism or event may be needed.

Among the four risk factors in multivariate analysis for development of SDF or CSDH, high-degree CSO-PVS, initial SDH presence, and old age (≥55 years) showed statistically significant effects on the development of SDF or CSDH over time. If the initial brain CT shows evidence of SDF or SDH in older patients with TBI, the prognosis of these patients can be predicted by confirming the severity of CSO-PVS through brain MRI, because it is likely to increase over time.

Recently, there have been many studies regarding the pathophysiology of CSDH development after TBI. Early theories suggested that SDF developed as a result of tears within bridging veins that traverse from the brain to the draining dural venous sinuses; by separation of the dura-arachnoid interface at the dural border cell layer; or by an arachnoid tear and CSF influx into the subdural space[39–41]. However, recent evidence suggests that there are more complex processes involved, because the pathophysiology of CSDH development cannot be explained in patients with no TBI, or in those who exhibit only mild TBI [39, 42]. Thus, the pathophysiology of the development of CSDH after any type of TBI may be influenced by complex interrelated mechanisms, including inflammation, impaired coagulation, fibrinolysis, and angiogenesis[39, 42]. Recently, Holl et al. described a pathway involved in the pathophysiology of CSDH after minor TBI, suggesting that cleavage of the dural border cell layer can activate cytokines, fibroblasts, and vascular endothelial growth factor. Finally, immature capillaries may allow extravasation into the hematoma cavity[42].

Our results have meaningful implications for predicting the prognosis of male patients ≥55 years of age with high-degree CSO-PVS after mild TBI. For patients with high-degree CSO-PVS, we can provide guidance regarding the length and frequency of monitoring through radiologic examinations.

This study had several limitations. First, selection bias was likely involved, as we included only patients who underwent T2-weighted MRI. Second, a limited number of patients required surgical treatment, which led to weak statistical power with regard to evaluating risk factors for surgery. Third, this was a retrospective study. Patients were treated with a variety of methods and had various follow-up periods. To overcome these limitations, a prospective study of mild TBI patients with high-degree CSO-PVS is need to confirm our findings and further evidence for guidance of clinical practice.

## Conclusions

High-degree CSO-PVS, male sex, initial SDF on admission, and old age were independently associated with development of de novo SDF. In addition, Cox proportional hazards models of risk factors affecting the duration of de novo SDH showed that high-degree CSO-PVS, old age, and initial SDH were statistically significant. Our study might help neurosurgeons determine the frequency of brain CT or the length of follow-up for patients who present with mild TBI with high-degree CSO-PVS. A large prospective study is warranted to validate our present findings.

## Supporting information

S1 File This is the S1 Excel file for data for submission. (XLS)
